# Supplementary material for: The Systems Biology Research Tool: evolvable open-source software
Source: BMC Syst Biol. 2008 Jun 29;2:55. doi: 10.1186/1752-0509-2-55 (PMC2446383; doi:10.1186/1752-0509-2-55)
Supplement: Additional file 1 — SBRT Archive. An archive of the current version of the Systems Biology Research Tool. [file 1752-0509-2-55-S1.zip › sbrt-1.4.0/doc/developers_guide/api/sbrt/kernel/fba/proc/package-frame.html]

sbrt.kernel.fba.proc


sbrt.kernel.fba.proc

Classes 
  
CatalystDeletionProcess
  
CdHarFluxVectorProcess
  
CvCatalystDeletionProcess
  
CvFbaOptimizationExperiment
  
CvFbaOptProcess
  
CvFluxIntervalProcess
  
CvObjectiveFunctionProcess
  
CvRxnDeletionProcess
  
CycleIdProcess
  
ExtremeCurrentProcess
  
FbaOptimizationProcess
  
FbaProcess
  
FbaSolverProcess
  
FluxActivityProcess
  
FluxActivityProcess.Results
  
FluxCapProcess
  
FluxIntervalProcess
  
FluxomeReducerProcess
  
FluxPlasticityProcess
  
FluxPlasticityProcess.Results
  
InitialPointGenerator
  
NetworkInfoProcess
  
NetworkInfoProcess.Results
  
ObjectiveFunctionAnalysis
  
RandomConstraintsGeneratorV1
  
RandomEnvGeneratorV1
  
RandomObjectiveGenerator
  
RxnDeletionProcess
  
StoichVariationProcess
  
SvCdHarFluxVectorProcess
  
SvInitialPointProcess
